# Barcoded monoclonal embryoids are a potential solution to confounding bottlenecks in mosaic organoid screens

**DOI:** 10.1101/2025.05.23.655669

**Published:** 2025-07-16

**Authors:** Samuel G. Regalado, Chengxiang Qiu, Jean-Benoît Lalanne, Beth K. Martin, Madeleine Duran, Cole Trapnell, Aidan Keith, Silvia Domcke, Jay Shendure

## Abstract

Genetic screens in organoids hold tremendous promise for accelerating discoveries at the intersection of genomics and developmental biology. Embryoid bodies (EBs) are self-organizing multicellular structures that recapitulate aspects of early mammalian embryogenesis. We set out to perform a CRISPR screen perturbing all transcription factors (TFs) in murine EBs. Specifically, a library of TF-targeting guide RNAs (gRNAs) was used to generate mouse embryonic stem cells (mESCs) bearing single TF knockouts. Aggregates of these mESCs were induced to form mouse EBs, such that each resulting EB was ’mosaic’ with respect to the TF perturbations represented among its constituent cells. Upon performing single cell RNA-seq (scRNA-seq) on cells derived from mosaic EBs, we found many TF perturbations exhibiting large and seemingly significant effects on the likelihood that individual cells would adopt certain fates, suggesting roles for these TFs in lineage specification. However, to our surprise, these results were not reproducible across biological replicates. Upon further investigation, we discovered cellular bottlenecks during EB differentiation that dramatically reduce clonal complexity, curtailing statistical power and confounding interpretation of mosaic screens. Towards addressing this challenge, we developed a scalable protocol in which each individual EB is monoclonally derived from a single mESC and genetically barcoded. In a proof-of-concept experiment, we show how these monoclonal EBs enable us to better quantify the consequences of TF perturbations as well as ’inter-individual’ heterogeneity across EBs harboring the same genetic perturbation. Looking forward, monoclonal EBs and EB-derived organoids may be powerful tools not only for genetic screens, but also for modeling Mendelian disorders, as their underlying genetic lesions are overwhelmingly constitutional ( *i.e.* present in all somatic cells), yet give rise to phenotypes with incomplete penetrance and variable expressivity.

## Full Text Availability

The license terms selected by the author(s) for this preprint version do not permit archiving in PMC. The full text is available from the preprint server.

