# Supplementary material for: Barcoded monoclonal embryoids are a potential solution to confounding bottlenecks in mosaic organoid screens
Source: bioRxiv. 2025 May 24:2025.05.23.655669. Preprint. [Version 1] doi: 10.1101/2025.05.23.655669 (PMC12139999; doi:10.1101/2025.05.23.655669)
Supplement: Supplementary file 1 [file NIHPP2025.05.23.655669V1-supplement-1.pdf]

bioRxiv preprint doi: https://doi.org/10.1101/2025.05.23.655669; this version posted May 24, 2025. The copyright holder for this preprint (which was not certified by peer review) is the author/funder, who has granted bioRxiv a license to display the preprint in perpetuity. It is made available under aCC-BY 4.0 International license.

Supplementary Tables

Supplementary Table 1. Marker genes used for mEB cell type annotation.

Supplementary Table 2. References highlighting the essential roles of 31 targets in stem cell and EB biology.

Supplementary Table 3. The gRNA library targeting genes or NTCs in each screening experiment.

Supplementary Table 4. Comparison of log2-scaled fold changes in gRNA frequencies between mESCs and the original plasmid library for CRISPR-cut and CRISPRi.

Supplementary Table 5. Comparison of gRNA frequencies between day 21 EBs and the original plasmid library.

Supplementary Table 6. Differentially expressed genes were identified by comparing cells with a targeted TF to NTCs in the lateral plate mesoderm.

Supplementary Table 7. Comparing cell abundances across 12 identified cell types between clonotypes assigned to each TF target and the NTC was performed using Hooke analysis.

Supplementary Table 8. Differentially expressed genes were identified using DESeq2 by comparing pseudobulk profiles of monoclonal EBs assigned to Hand1 and NTC, reporting only results with adjusted p-values <0.05.

bioRxiv preprint doi: https://doi.org/10.1101/2025.05.23.655669; this version posted May 24, 2025. The copyright holder for this preprint (which was not certified by peer review) is the author/funder, who has granted bioRxiv a license to display the preprint in perpetuity. It is made available under aCC-BY 4.0 International license.

SUPPLEMENTARY FIGURES

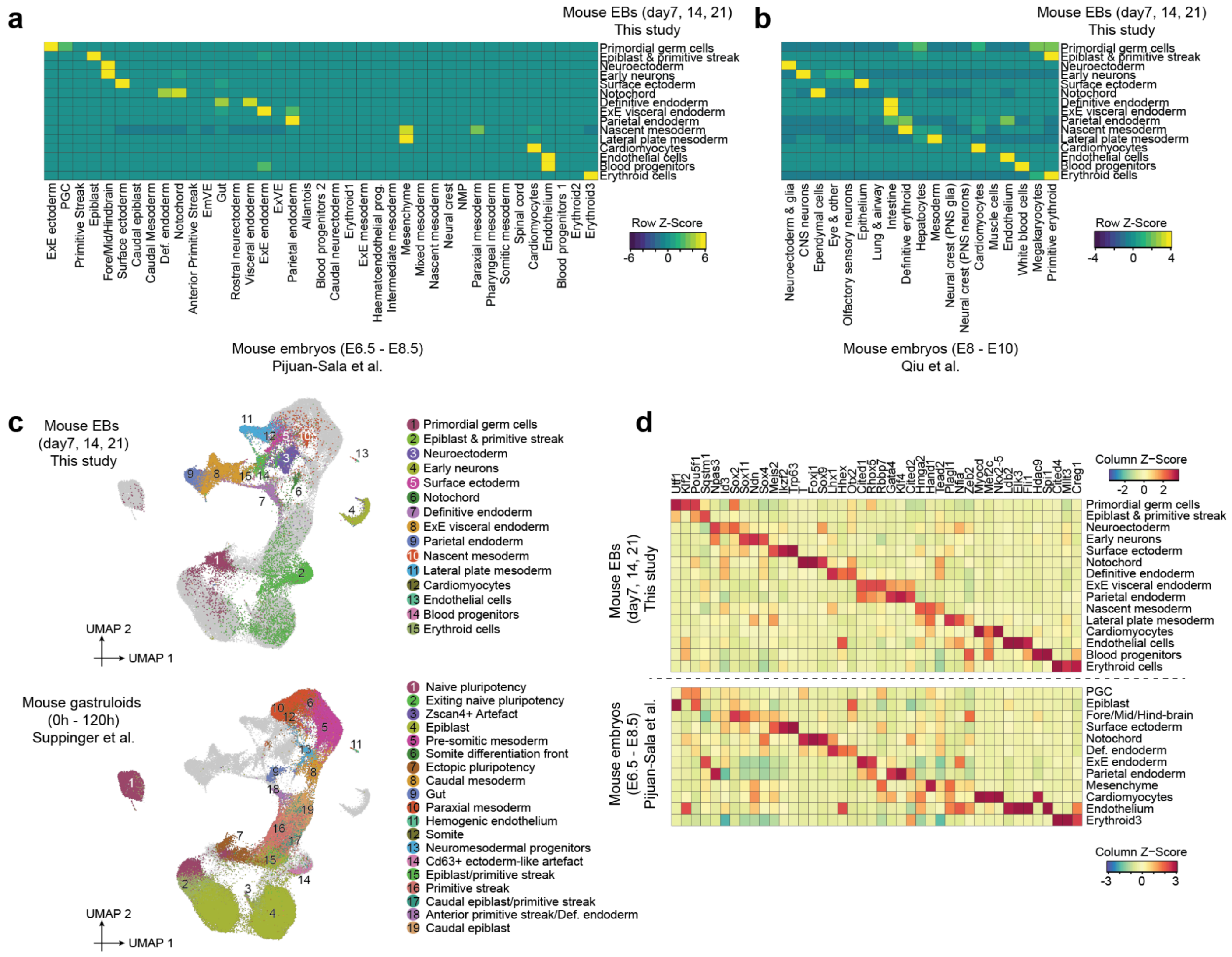

Supplementary Figure 1. Integrating and co-embedding cells from mouse EBs, embryos, and gastruloids. a, Correlated developmental major cell clusters between mouse embryos during gastrulation[23] (column) and mouse EBs (row) based on non-negative least-squares (NNLS) regression. Heatmap shows the combined regression coefficients (row-scaled) of pairwise cell types between the two datasets. **b,** Correlated developmental major cell clusters between mouse embryos during early somitogenesis[24] (column) and mouse EBs (row) based on non-negative least-squares (NNLS) regression. Heatmap shows the combined regression coefficients (row-scaled) of pairwise cell types between the two datasets. **c,** UMAP visualization of co-embedded cells from mouse EBs and gastruloids[25] after batch correction of scRNA-seq data. The same UMAP is shown twice, with colors highlighting cells from either mouse EBs (top) or gastruloids (bottom). **d,** Expression profiles of the top 3 TF markers of the 15 clusters shown in **Fig. 1a**, as identified using the FindAllMarkers function of Seurat/v3[66], within mouse EBs (top) or mouse embryos during gastrulation[23] (bottom). Each heatmap illustrates the mean gene expression values within each cluster, calculated from original UMI counts normalized to total UMIs per cell, followed by natural-log transformation. For each cell cluster from the EB dataset, the most similar cell type from mouse embryos was manually selected based on top marker genes, complemented by cell-type correlation analysis, as shown in panel **a**. Overall, we observe that the cell type-specific expression patterns of many of these TFs are shared between related cell types in mouse EBs and gastrulating mouse embryos.

bioRxiv preprint doi: https://doi.org/10.1101/2025.05.23.655669; this version posted May 24, 2025. The copyright holder for this preprint (which was not certified by peer review) is the author/funder, who has granted bioRxiv a license to display the preprint in perpetuity. It is made available under aCC-BY 4.0 International license.

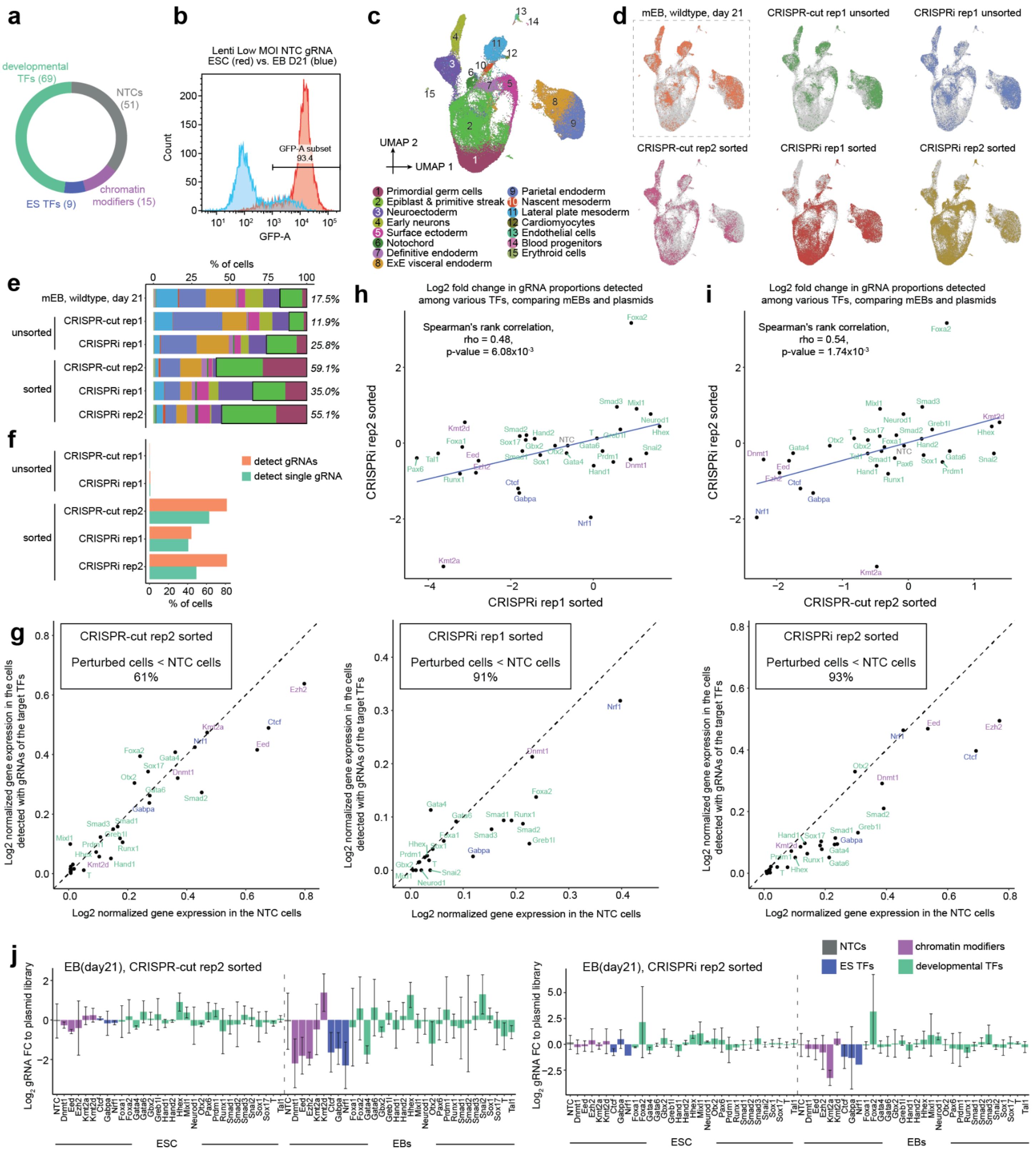

Supplementary Figure 2. Quality assessment of the CRISPRcut and CRISPRi systems in mouse EBs ('pilot-mosaic' experiment). a, Pie-chart of pilot library composition. In the pilot screen, a total of 144 gRNAs were designed. Among these, 51 gRNAs corresponded to non-targeting controls (NTC), while the remaining 93 gRNAs were specific to 31 target genes, with 3 gRNAs allocated per target. Target genes were selected to include 3 TFs thought to be essential in mESCs, 5 chromatin modifiers thought to be non-essential in mESCs but required for EB differentiation and 23 TFs with reported roles in germ layer formation. **b,** Exemplary FACS analysis of mESCs (here: CRISPRcut mESCs)

bioRxiv preprint doi: https://doi.org/10.1101/2025.05.23.655669; this version posted May 24, 2025. The copyright holder for this preprint (which was not certified by peer review) is the author/funder, who has granted bioRxiv a license to display the preprint in perpetuity. It is made available under aCC-BY 4.0 International license.

transduced with a lentiviral CROPseq library (here: NTC gRNAs), and of 21 day-old EBs generated from these same mESCs, showing loss of GFP expression over the course of differentiation (here: 93% → 21%). Similar ratios were observed for all libraries and cell lines. **c,** 2D UMAP visualization of scRNA-seq profiles from 66,213 co-embedded cells derived from wildtype or perturbed mouse EBs at day 21, following batch correction. These perturbed samples originate from four pilot experiments conducted on mouse EBs at day 21, utilizing either CRISPRcut or CRISPRi techniques, with sorting for GFP-positive cells or without and with two different transduction rates: CRISPRcut rep1 sorted and unsorted (1% GFP positive; not enough cells recovered for sorted population), CRISPRcut rep2 sorted (<20% GFP positive), CRISPRi rep1 sorted and unsorted (1% GFP positive), CRISPRi rep2 sorted (<20% GFP positive). **d,** The same UMAP as in panel **c** is shown multiple times, with colors highlighting cells derived from either wildtype EBs or perturbed EBs from each pilot experiment. **e,** Cell cluster composition of mouse EBs, either from wildtype EBs or perturbed EBs from each pilot experiment. The percentage of less differentiated cells (primordial germ cells, and epiblast & primitive streak) is shown on the right side of each bar. **f,** The percentage of cells with at least one gRNA captured, as well as those with a unique gRNA captured, is reported for each experiment. **g,** Log2-normalized gene expression levels of a particular gene were compared between cells with NTC gRNAs and cells with gRNAs specifically targeting that gene, in three pilot experiments. The percentage of genes with lower expression in perturbed cells compared to NTC cells (below the y = x dotted line) is highlighted in each panel. **h,** Comparison of the log2-fold-change in the proportion of gRNAs targeting a specific gene or NTC between mouse EBs and plasmid library, across two independent CRISPRi pilot experiments. **i,** Comparison of the log2-fold-change in the proportion of gRNAs targeting a specific gene or NTC between mouse EBs and plasmid library, across CRISPRcut and CRISPRi pilot experiments. **j,** Changes in gRNA frequency for different target genes. The log2-fold-change in the proportion of gRNAs was compared between mESCs and plasmid library, or between EBs and plasmid library, in both the CRISPRcut pilot experiment (left) and the CRISPRi pilot experiment (right). Bars represent the average log2-fold-change of gRNAs targeting each gene, with error bars indicating the mean ± standard deviation. In panels **g-j**, target genes are categorized into chromatin modifiers, ES TFs, or developmental TFs.

bioRxiv preprint doi: https://doi.org/10.1101/2025.05.23.655669; this version posted May 24, 2025. The copyright holder for this preprint (which was not certified by peer review) is the author/funder, who has granted bioRxiv a license to display the preprint in perpetuity. It is made available under aCC-BY 4.0 International license.

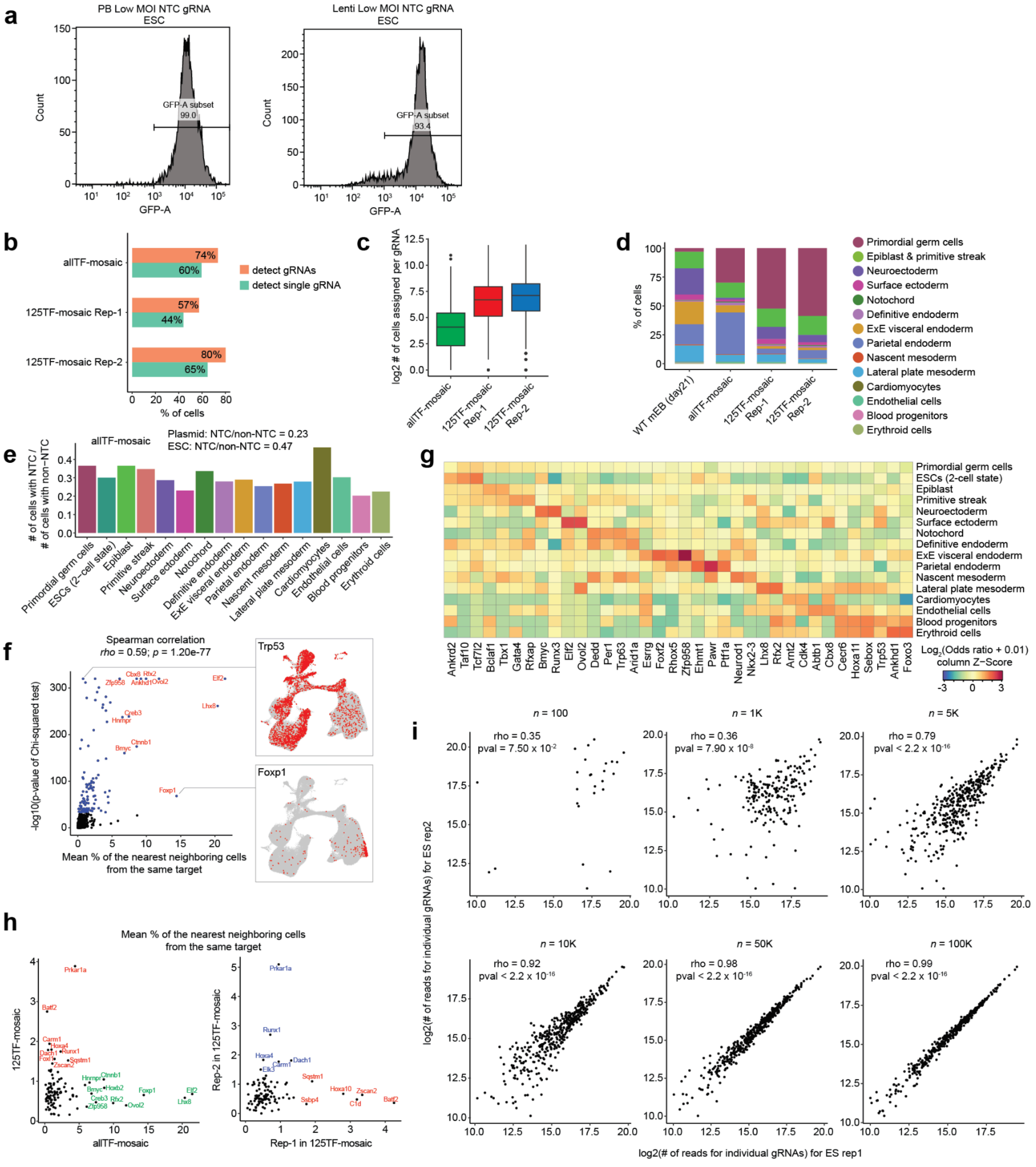

Supplementary Figure 3. Quality-control of large-scale TF screens in mosaic EBs. a, FACS analysis showing high rate of GFP-positive (*i.e.* inferred to be gRNA-expressing) cells after applying puro selection to a starting cell population with <10% GFP-positive cells post-transduction. Due to the combination of low transduction rate and drug selection, the resulting cell population is expected to consist almost entirely of cells expressing exactly one gRNA. **b,** The percentage of cells with at least one gRNA captured, as well as those with a unique gRNA captured, is reported for each of three TF screen datasets. **c,** The number of cells assigned per gRNA were reported for each of the three TF screen datasets (*n* =

bioRxiv preprint doi: https://doi.org/10.1101/2025.05.23.655669; this version posted May 24, 2025. The copyright holder for this preprint (which was not certified by peer review) is the author/funder, who has granted bioRxiv a license to display the preprint in perpetuity. It is made available under aCC-BY 4.0 International license.

4,931 gRNAs for ‘allTF-mosaic’ experiment, and *n* = 433 and 435 for replicates 1 and 2, respectively, of ‘125TF-mosaic’ experiment). Boxplots represent IQR (25th, 50th, 75th percentile) with whiskers representing 1.5× IQR. **d,** Composition of mouse EBs, either from wildtype EBs (21 days) or perturbed EBs from each of the three TF screen datasets, binned by cell cluster. Early neurons, floor plate, and eye field were merged into 'neuroectoderm', while epiblast, primitive streak, and ESCs (2-cell state) were merged into 'epiblast & primitive streak' to align with classifications across the different datasets. **e,** Ratio of cells assigned to NTC gRNA versus non-NTC gRNA for each cell type (‘allTF-mosaic’ experiment). **f,** From the ‘allTF-mosaic’ dataset, TF targets with gRNAs detected in fewer than 50 cells were excluded, and cells with each TF target were randomly downsampled to 200 cells. Individual cells were searched for 20-nearest neighbors in PCA space (*n* = 30 dimensions). For each TF target, the average proportion of nearest neighbor cells sharing the same TF target was calculated. These average proportions (x-axis) were then compared with the p-values obtained from chi-squared tests (y-axis) for the same TF targets. Significant TF targets identified by the chi-squared tests are highlighted in blue. Selected TF targets with high values on both axes are labeled. The same UMAP as in **Fig. 2a** is shown twice on the right, with colors highlighting cells where *Trp53* or *Foxp1* transcripts were detected. **g,** From the ‘allTF-mosaic’ dataset, TF targets with gRNAs detected in fewer than 50 cells were filtered out. For each remaining TF target and each of the 16 cell clusters, we performed Fisher’s exact test on the frequency of cells within or outside the cell cluster between cells with the TF target and all cells with NTC gRNAs. The $\log_2$(odds ratio + 0.01) of the top three TF targets (ranked by p-value) for each cell cluster is presented in the heatmap. **h,** Comparison between experiments of proportion of cells’ nearest neighbors in PCA space sharing the same target TF. For each dataset and for each TF target, the average proportion of nearest neighbor cells within the 20-nearest neighbors in PCA space that shared the same TF target was calculated. Left: comparison of ‘allTF-mosaic’ vs. combined replicates of ‘125TF-mosaic’ experiments. Right: comparison of two replicates of ‘125TF-mosaic’ experiment. Selected TF targets with high proportions in either experiment being compared are labeled. **i,** Standard curve assessing our ability to quantify gRNA read frequency from genomic DNA for different starting cell numbers. Two replicates of variable numbers of mESCs (100, 1K, 5K, 10K, 50K, 100K) containing the 125 TF library (456 distinct gRNAs, with mostly 1 gRNA/cell) were sampled, their genomic DNA was isolated, the gRNA amplified and sequenced. This experiment verified our ability to reproducibly quantify gRNA frequency from as few as 10,000 cells (R=0.92). This serves as a control for quantifying gRNA frequencies from genomic DNA isolated from EB cells containing the same library (each EB contains at least 10K cells, but typically around 30K).

bioRxiv preprint doi: https://doi.org/10.1101/2025.05.23.655669; this version posted May 24, 2025. The copyright holder for this preprint (which was not certified by peer review) is the author/funder, who has granted bioRxiv a license to display the preprint in perpetuity. It is made available under aCC-BY 4.0 International license.

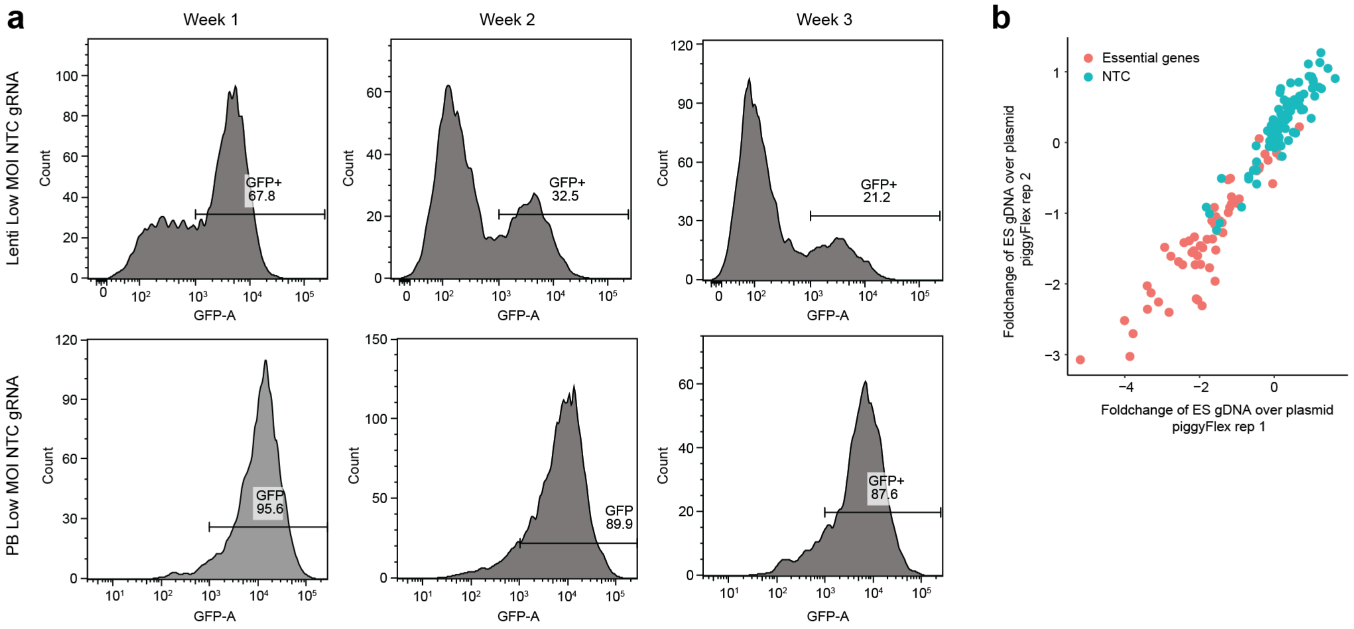

Supplementary Figure 4. Validation of stable expression during differentiation and functionality of gRNAs with the integrated piggyFlex construct. a, FACS analysis plots showing different rates of loss of GFP expression over the course of one, two or three weeks of EB differentiation, for lentiviral CROP-seq vectors (top) and transposon-based piggyFlex (bottom). **b,** Reproducible activity of piggyFlex construct in CRISPRcut mESCs. Fourteen days after transfecting cells with a piggyFlex library of gRNAs containing NTCs and gRNAs targeting essential genes, genomic DNA was isolated and the contained gRNAs amplified by PCR from both the genomic DNA and the plasmid library. Reproducible depletion of gRNAs targeting essential genes (red) compared to NTCs (green) in genomic DNA from two independently transfected replicates implies the CRISPRcut-piggyFlex system is functional.

bioRxiv preprint doi: https://doi.org/10.1101/2025.05.23.655669; this version posted May 24, 2025. The copyright holder for this preprint (which was not certified by peer review) is the author/funder, who has granted bioRxiv a license to display the preprint in perpetuity. It is made available under aCC-BY 4.0 International license.

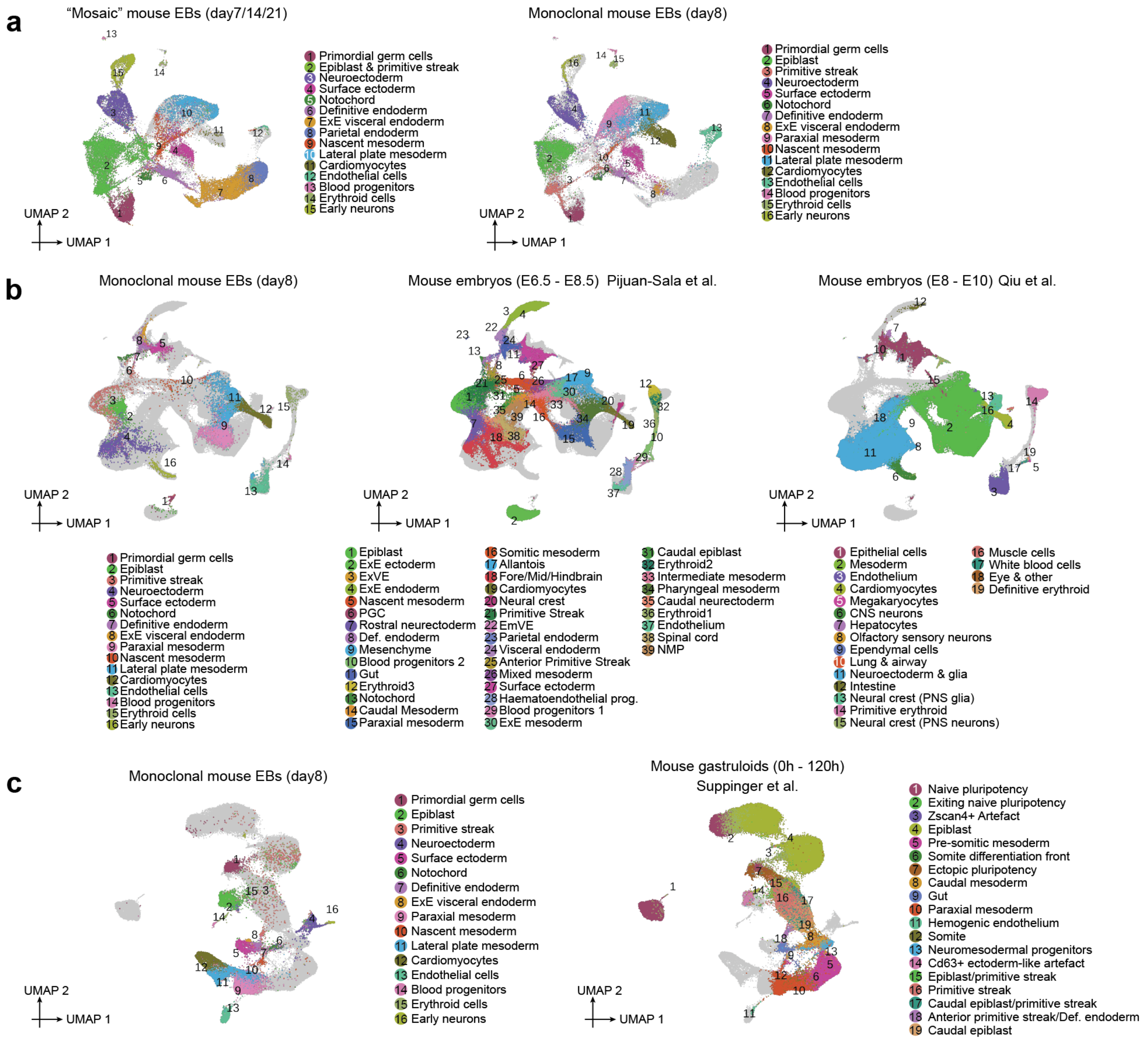

Supplementary Figure 5. Integrating and co-embedding cells from monoclonal mouse EBs, embryos, and gastruloids. a, UMAP visualization of co-embedded cells from 'mosaic' mouse EBs and monoclonal mouse EBs after batch correction of scRNA-seq data. The same UMAP is shown twice, with colors highlighting cells from either 'mosaic' mouse EBs (left), or monoclonal mouse EBs (right). **b,** UMAP visualization of co-embedded cells from monoclonal mouse EBs and real embryos at various developmental stages after batch correction of scRNA-seq data. The same UMAP is shown three times, with colors highlighting cells from either mouse EBs (left), embryos during gastrulation[23] (middle), or embryos during early somitogenesis[24] (right). **c,** UMAP visualization of co-embedded cells from monoclonal mouse EBs and gastruloids after batch correction of scRNA-seq data. The same UMAP is shown twice, with colors highlighting cells from either mouse EBs (left) or gastruloids[25] (right).
